# Associations Between Maternal Cumulative Psychological Distress and Child Subsequent Social Competence: The Role of Early Childhood Education and Care and Social Networks

**DOI:** 10.1111/sjop.70087

**Published:** 2026-03-20

**Authors:** Katja Tervahartiala, Eeva‐Leena Kataja, Laura Perasto, Niina Junttila, Marjukka Pajulo, Hasse Karlsson, Noona Kiuru, Saara Nolvi, Linnea Karlsson, Riikka Korja

**Affiliations:** ^1^ Department of Psychology and Speech‐Language Pathology University of Turku Turku Finland; ^2^ Department of Psychology University of Jyväskylä Jyväskylä Finland; ^3^ Centre of Excellence in Learning Dynamics and Intervention Research (InterLearn), University of Jyväskylä and University of Turku Jyväskylä Finland; ^4^ Department of Clinical Medicine FinnBrain Birth Cohort Study, Turku Brain and Mind Center University of Turku and Turku University Hospital Turku Finland; ^5^ Centre for Population Health Research University of Turku and Turku University Hospital Turku Finland; ^6^ Department of Teacher Education University of Turku Turku Finland; ^7^ Department of Teacher Education University of Jyväskylä Jyväskylä Finland; ^8^ Department of Child Psychiatry University of Turku and Turku University Hospital Turku Finland; ^9^ Department of Psychiatry University of Turku and Turku University Hospital Turku Finland; ^10^ Department of Clinical Medicine, Unit of Public Health University of Turku and Turku University Hospital Turku Finland

**Keywords:** antisocial behavior, early childhood education and care, prosocial behavior, psychological distress, social networks

## Abstract

Maternal psychological distress may have adverse effects on child socioemotional development. However, supportive social networks and participation in out‐of‐home childcare may serve as key protective factors and promote positive developmental outcomes. This study investigated whether maternal cumulative psychological distress is associated with children's (*n* = 528) social competence at the age of 5 years (*M* = 5.02, SD = 0.08) in Finland. Latent profile analyses (LPA) were conducted to identify latent classes of maternal cumulative symptoms of depression, anxiety, and parenting stress. Associations between these profiles and children's prosocial and antisocial behavior were examined. Additionally, the moderating effects of the child's age at entry into Early Childhood Education and Care (ECEC) and maternal supportive social networks were analyzed. The results showed that chronically high maternal psychological distress was subsequently associated with child's higher impulsivity, higher disruptiveness, and lower empathy. We found no evidence of early ECEC attendance or mother's social networks as moderators between maternal symptoms and child's behavior. Nevertheless, mother's supportive social networks were beneficial for all children and associated with child's higher empathy and cooperation skills. These findings underscore the need for early identification of maternal psychological distress symptoms. Moreover, social networks may serve as valuable resources for families with young children and support child development, even though they did not buffer the effects of maternal distress.

## Summary

- Chronically high maternal psychological distress was associated with a child's higher impulsivity, higher disruptiveness, and lower empathy at the age of 5 years.
- Early start in ECEC or mother's social networks did not buffer the effects of maternal distress symptoms on child socioemotional outcomes.
- Maternal supportive social networks were beneficial for all children and associated with a child's higher empathy and better cooperation skills.
- Social networks constitute significant resources for families with young children, particularly when parents' own resources are limited.
- It is important to foster cooperative infrastructure, alleviate loneliness, and support diverse social networks for families with young children.

## 1 Introduction

Family environment is the primary social and emotional context for young children. Parental well‐being plays a crucial role in shaping parenting behavior, parent–child interactions, and child development (Goodman et al. 2011). There is strong evidence indicating that maternal psychological distress, such as parenting stress and depressive and anxiety symptoms, can compromise children's socioemotional development and is associated with adverse child outcomes (Glasheen et al. 2010; Goodman et al. 2011; Östberg and Hagekull 2013). A recent review by Morales et al. (2023) indicated that chronic and persistent maternal symptoms, in particular, have the most significant impact on child development and should be targeted with supportive interventions. Given the high prevalence estimates, ranging from 10% to 40% of various maternal distress symptoms during postnatal years (Field 2018; O'Hara and McCabe 2013), it is essential to explore how these symptoms co‐occur, accumulate, and influence children's developmental outcomes.

The present study explored cumulative profiles of maternal self‐reported anxiety and depressive symptoms and parenting stress. Although these symptoms have somewhat distinct presentations and backgrounds, they may co‐occur and accumulate, potentially exerting an even more detrimental effect on child development. Previous research has consistently demonstrated comorbidity between various types of psychological distress symptoms (Falah‐Hassani et al. 2017; Kerstis et al. 2016). The systematic review and meta‐analysis of Falah‐Hassani et al. (2017) showed that maternal depressive and anxiety symptoms co‐occurred both during pregnancy and postnatal periods. Also, parents experiencing depressive symptoms tend to report higher levels of parenting stress compared to those without such symptoms. This suggests that various forms of psychological distress often overlap among parents (Kerstis et al. 2016; Lim and Shim 2021).

Typical symptoms of anxiety include constant worry, restlessness, and difficulties in concentrating and sleeping (Vanin 2008; Derogatis et al. 1973). Depression, in turn, may present as sadness, feelings of hopelessness, and loss of interest and pleasure (Zimmerman et al. 2018; Cox et al. 1987). Parenting stress is a specific form of stress arising from the high demands and limited resources associated with parenting. It may result from non‐optimal family functioning, lack of social support, caregiving challenges, child behavioral problems, health issues, or relationship difficulties (Louie et al. 2017; Östberg et al. 1997). Psychological distress symptoms typically have adverse effects on quality of life, family functioning, and parenting behavior (Hohls et al. 2021; Dong et al. 2022; Jones et al. 2021). Despite the reported similarities in the effects of depression, anxiety, and parenting stress on child socioemotional development, prior research suggests that these symptoms may also have somewhat different impacts on caregiving behavior and child outcomes. Maternal anxiety symptoms have been associated with higher levels of maternal intrusiveness (Hakanen et al. 2019), overprotective parenting (Jones et al. 2021), and lower levels of warmth and involvement (Seymour et al. 2015). Maternal depressive symptoms, in turn, have been linked to reduced sensitivity (Feldman et al. 2009), increased irritability and hostility toward the child, as well as less positive interaction and play (Lovejoy et al. 2000). Parenting stress may lead to harsher, more negative, and less stimulating interactions with a child (Liu et al. 2020).

Because parental psychological distress symptoms may have different forms and diverse effects, it is important to examine how these symptoms co‐occur and accumulate among parents. The most concerning condition is characterized by the overlapping effects of chronic and multiple types of symptoms on children's socioemotional development.

### 1.1 The Development of Social Competence in Early Childhood

Socioemotional development is a broad concept that encompasses social competence, including the aspects of both prosocial and antisocial behavior. Social competence undergoes rapid development beginning in toddlerhood and continuing throughout childhood (Santos et al. 2014). Previous research has shown that both individual child characteristics and environmental factors, as well as the interaction between them, shape the development of social competence (Behrendt et al. 2020). In the present study, social competence is examined through both prosocial and antisocial aspects of children's social behavior. Prosocial behavior refers to socially desirable actions, such as helping, sharing, and comforting, and is composed of the dimensions of cooperation skills and empathy (Eisenberg and Spinrad 2014; Junttila et al. 2006). These actions are valued by society and are actively encouraged in children. Prosocial behaviors, such as cooperating and participating in group activities, contribute to peer acceptance and support learning and academic achievements (Kochenderfer‐Ladd and Ladd 2019). In contrast, difficulties in prosocial behavior may present as antisocial behavior, including impulsive or disruptive actions. Antisocial behavior is associated with negative social outcomes; it may be either intentional or unintentional and can be directed toward others or oneself. To be socially competent, a child typically behaves strongly on the dimension of prosocial behavior and low on the dimension of antisocial behavior (Junttila et al. 2006).

### 1.2 The Role of Environmental Protective Factors in Supporting the Development of Social Competence

In addition to risk factors, it is important to consider environmental protective factors that support the development of social competence. In this study, we aimed to identify environmental factors that may directly or indirectly support parenting and promote the development of child social competence. Bronfenbrenner's (1979) ecological systems theory provides a structured framework for examining how various environmental factors contribute to a child's development. Microsystem refers to a child's closest relationships with caregivers in a family context and in out‐of‐home childcare settings, while mesosystem refers to communication between these early childhood environmental settings. Exosystem, in turn, encompasses a wider social network such as neighbors, friends, and community. The development is also a result of continuous and bidirectional interaction between a child and the rearing environment. A child's individual characteristics influence how the environment responds to the child's initials and vice versa (Sameroff 2009). Previous research suggests that emotional problems, in particular, may show bidirectional associations between parents and their children, whereby each influences the other's behavior and emotions (Verhagen et al. 2026; Lowthian et al. 2023). However, the present study was not able to examine such bidirectional processes, and it focused solely on maternal influences on child socioemotional development.

Family environment in the microsystem level is the primary rearing environment for the youngest children. Later, Early Childhood Education and Care (ECEC) plays an important role for most children in industrialized societies (OECD 2024) and may serve as a protective factor when there are risks present within the family (Berry et al. 2016). In particular, when parental distress or a chaotic home environment is present, participation in ECEC may play an important buffering role against these adverse family conditions (Charrois et al. 2017). In such settings, children may benefit from sensitive interaction with teachers and enhanced support for social and emotional development, especially when parenting resources at home are limited (Berry et al. 2016). Positive communication and collaboration between parents and teachers in ECEC may facilitate a supportive environment for a child and improve parental resources.

Nonetheless, for ECEC to effectively fulfill this protective role, sufficient resources are required, and early childhood educators must be appropriately trained and competent to support children from diverse backgrounds with varying needs. However, it is important to note that while ECEC may alleviate disadvantages, it may also contribute to cumulative risk if the quality of care is insufficient. Recent findings by Parkes et al. (2021) suggest that children facing multiple adversities, such as poor maternal mental health and economic hardship, benefit less from ECEC compared to children with fewer environmental risks. In contrast, most studies conducted in Nordic countries have reported positive associations between ECEC attendance and children's developmental outcomes (Esping‐Andersen et al. 2012; Wahler et al. 2017). High‐quality ECEC serves as a valuable learning environment for preparing children to achieve social and emotional competencies that are essential for both peer relationships and academic success.

Additionally, in Finland, where the present study was conducted, special support within ECEC is provided for children with developmental deficits or multiple risk factors in their home environment (Äikäs et al. 2023). Finnish ECEC is highly regulated by the National Agency for Education, resulting in a more consistent quality of formal ECEC compared to many other countries (Kulic et al. 2017). Regulations cover group sizes, staff‐to‐child ratios, and teacher qualifications (Minedu 2025). Given this structured and supportive system, participation in ECEC should be considered a potential protective factor when examining the effects of parental psychological distress on children's developmental outcomes.

Another important protective factor in child development is the social networks of the family. These factors in the exosystem‐level may have indirect influences on child development. Beneficial social networks refer to connections with friends, relatives, and neighbors, as well as the support received in caring for the child (Östberg et al. 1997). Recent findings by Neitola et al. (2023) highlight the significant role of parental social networks in children's peer relationships. Specifically, mothers' social networks were positively associated with the size of children's own social networks and the number of friends the child had. Moreover, children's peer networks and number of social connections appear to show some stability across childhood (Neitola et al. 2023). Social networks and support are particularly important when parents experience high levels of parenting stress (Hong and Liu 2021). Elevated parenting stress, especially in combination with other stressors, has been linked to less appropriate parenting practices and increased parent–child conflicts. However, social support can buffer these effects, promoting more positive parenting behaviors (Liu et al. 2020). Previous research has also shown that the association between parenting stress and depressive symptoms is weaker among mothers with stronger social networks and resources, highlighting the protective role of social support for parental mental health (Nagy et al. 2022). Hence, social contacts and support can enhance parental well‐being, foster more sensitive caregiving, and positively influence child development.

Given that many different global and societal factors currently impose stress on families and may have adverse effects on parental mental health, it is crucial to investigate environmental protective factors that promote effective parenting and foster child socioemotional development.

Working in this context, the specific research questions (RQs) of the present study were as follows:

RQ1. To investigate whether maternal cumulative psychological distress, including parenting stress and depressive and anxiety symptoms (from 3 months to 4 years of age), is associated with prosocial behavior, such as cooperation skills and empathy, and antisocial behavior, such as disruptiveness and impulsivity, at the child age of 5 years.

RQ2. To analyze the associations between the age at which the child entered ECEC, the mother's social networks, and the child's prosocial and antisocial behavior at the age of 5 years.

RQ3. To examine whether maternal social networks and the age at which the child entered ECEC moderate the potential associations between maternal psychological distress and child prosocial and antisocial behavior at the age of 5 years.

## 2 Materials and Methods

### 2.1 Participants and Procedure

The participants were drawn from the FinnBrain Birth Cohort Study (*N* = 3808), which is a large multidisciplinary population‐based longitudinal cohort study that was launched in 2010 in Finland. The aim of the FinnBrain is to study prenatal and early‐life stress and its associations with child development and later health (Karlsson et al. 2018). The birth cohort study consists of many sub‐studies including different study visits that cover various aspects of child development. The families in the present study (*n* = 532) participated in the Child Development and Parental Functioning Lab study visit, which consisted of different psychological tests and assessments. A total of 63.5% of the participants belonged to the “Focus Cohort” that consisted of children exposed to high versus low levels of prenatal maternal distress, but the population was also enriched by other actively participating families. Mothers completed the Multisource Assessment of Children's Social Competence (MASCS) questionnaire on their child's social competence at the age of 5 years and questionnaires on their parenting stress and depressive and anxiety symptoms at different age points along with the cohort data collection, from the age of 3 months to 4 years. The MASCS score was missing for four participants; therefore, the final number of participants in the current study was 528. All study participants gave their written informed consent, and parents gave consent on behalf of their children.

### 2.2 Measures

Parenting stress was measured by the maternal report of the Swedish Parenthood Stress Questionnaire (SPSQ) (Östberg et al. 1997; Östberg and Hagekull 2013) at the child's ages of 3 and 12 months. The SPSQ consists of 34 questions that are scored on a 5‐point Likert‐type scale. The SPSQ has a Total Parenting Stress Scale and five subscales: Incompetence, Role Restriction, Social Isolation, Spouse Relationship Problem, and Health Problems. Only the Total Parenting Stress Scale was used in the present study (*α* = 0.89–0.90).

Mothers' depressive symptoms were measured by the maternal report of the Edinburgh Postnatal Depression Scale (EPDS) (Cox et al. 1987) at the child's ages of 3, 6, and 12 months and 2 and 4 years. The EPDS consists of 10 items that are scored from 0 to 3, and higher total scores indicate higher levels of depression (*α* = 0.82–0.86). Mothers' anxiety symptoms were measured using the Symptom Checklist‐90 (SCL‐90) (Derogatis et al. 1973) at the child's ages of 3 and 6 months, and 2 and 4 years. The questionnaire has 10 items scored from 0 to 5, with higher scores reflecting higher levels of anxiety symptoms (*α* = 0.80–0.88).

Child social competence was evaluated at the age of 5 years using the maternal report of the Multisource Assessment of Children's Social Competence (MASCS), which has been validated in the Finnish context (Junttila et al. 2006). The MASCS measures a child's behavior in different social contexts and has 15 items that are scored on four options ranging from “never” to “very often.” The MASCS has two main dimensions: prosocial (*α* = 0.84) and antisocial (*α* = 0.82) aspects of social behavior. These main dimensions are further divided into four sub‐dimensions: cooperating skills (*α* = 0.79) and empathy (*α* = 0.74) (forming the prosocial dimension) and impulsivity (*α* = 0.84) and disruptiveness (*α* = 0.80) (forming the antisocial dimension).

Mothers' social networks and received support were measured using the self‐constructed questions that were based on the previous literature of supportive social networks (e.g., Kalland et al. 2022). Five questions measured how often the mother has the opportunity to meet her friends and relatives and neighbors and receive help for childcare from child's grandparents and the other parent (Table 2). The score has five options ranging from “not at all” … to “very often,” and these questions were measured at the child's ages of 3 and 12 months and 4 years. The 4‐year data were primarily used in the statistical analysis. Because the different measurement points were correlated (*r* 0.33–0.69), the missing values in the 4‐year measurement were replaced with 12‐month and 3‐month measurements.

Information on the child's age when entering ECEC was collected from the FinnBrain Cohort Study questionnaires at 12 months and 2 years of age and later by personal contacts by the research nurses. Maternal level of education was obtained from the birth cohort questionnaires, and children's non‐verbal cognitive ability (performance intelligence; PIQ) was assessed using the Wechsler Preschool and Primary Scale of Intelligence‐Third Edition (Wechsler 1991) during the study visit. The PIQ and maternal education were controlled for in the statistical analysis because earlier research has shown that maternal education (Huang et al. 2022) and children's non‐verbal cognitive ability (Tong et al. 2010) may be associated with children's socioemotional development.

### 2.3 Data Analyses

The latent profile analyses (LPA) were performed using Mplus software version 6 (Muthén and Muthén 2010). First, a longitudinal confirmatory factor analysis was conducted to analyze the validity and stability of the SPSQ, EPDS, and SCL‐90 scores across the measurement points. The fit indices estimated a good fit for each measure: EPDS 𝜒^2^ = 14.850 (df = 5); CFI = 0.96; TLI = 0.91; RMSEA = 0.07, 90% CI [0.03, 0.12]; SRMR = 0.04; SCL‐90 𝜒^2^ = 1.303 (df = 2); CFI = 1.0; TLI = 1.0; RMSEA = 0.00, 90% CI [0.00, 0.10]; SRMR = 0.01; SPSQ 𝜒^2^ = 20.89 (df = 1); CFI = 0.95; TLI = 0.96; RMSEA = 0.06, 90% CI [0.02, 0.11]; SRMR = 0.05.

**FIGURE 1:**
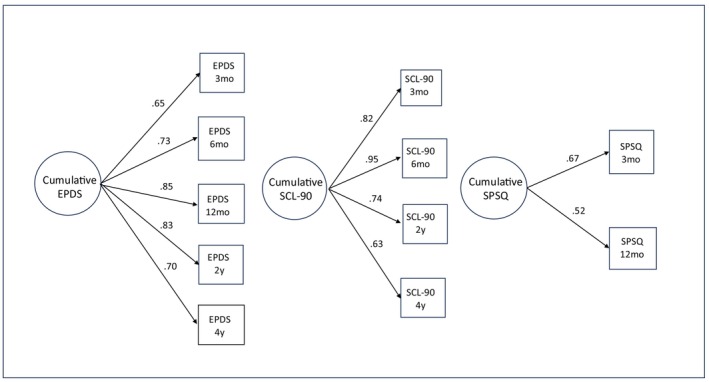
Cumulative latent variables and factor loadings of EPDS, SCL‐90, and SPSQ measures.

The aim was to examine mothers' experiences of parenting stress, anxiety, and depressive symptoms as a cumulative experience, specifically, the combined levels of these symptoms across multiple measurement points. Consequently, cumulative latent variables were constructed from the above‐mentioned variables measured at different time points. Accordingly, cumulative latent variables were modeled based on (1) the SPSQ at 3 and 12 months of age, (2) the EPDS at 3, 6, 12, 24, and 48 months of age, and (3) the SCL‐90 at 3, 6, 24, and 48 months of age (Figure 1).

Finally, a latent profile analysis (LPA) was used to identify the possible latent classes of cumulative maternal distress. We fitted LPA models with increasing numbers of groups to the data, and used log‐likelihood, the Akaike information criterion (AIC), the Bayesian information criterion (BIC), and Vuong‐Lo–Mendell–Rubin likelihood ratio tests to compare the models with different consecutive numbers of profiles. Smaller AIC and BIC estimates indicated a better model fit (Geiser 2013), and an entropy value above 0.80 indicated the distinctness and reliability of the latent classes (Rost 2006). Moreover, a significant *p*‐value (< 0.05) in the Vuong–Lo–Mendell–Rubin likelihood ratio test indicated that the model fitted the data better than a model with one fewer group (Nylund et al. 2007). We also acknowledged latent class size, interpretability, and theoretical justification when choosing between the models (Muthén 2003). The LPA model fit statistics are presented in Table 1. Based on fit indices and theoretical interpretation, a two‐class solution was selected for further analyses. In the three‐class solution, one of the classes was quite small (only 8% of parents) and the Entropy value dropped after the two‐class solution. Also, the Vuong–Lo–Mendell Test guided us to choose either a two or four class solution. However, for the four‐class solution, the Entropy value was smaller than for the two class solution and two classes had less than 10% of mothers. The estimated mean scores for the two class solution were 4.42 for EPDS; 5.52 for SCL‐90; and 0.51 for SPSQ.

**TABLE 1:** Statistics for LPA model fit.

PSE model	Log likelihood	AIC	BIC	Class proportions	Entropy	Average latent class posterior probabilities	Vuong–Lo–Mendell Test
1 Class	−2827.61	5667.22	5692.88	1.00	1.0	1.0	n/a
2 Class	−2528.93	5667.22	5120.63	0.87/0.13	0.95	0.99/0.96	< 0.00001
3 Class	−2415.32	4858.63	4918.50	0.29/0.63/0.08	0.86	0.90/0.95/0.96	0.22
4 Class	−2343.41	4722.83	4799.81	0.58/0.03/0.29/0.09	0.88	0.94/0.99/0.90/0.94	0.0003
5 Class	−2322.17	4688.34	4782.42	0.57/0.07/0.29/0.04/0.03	0.90	0.95/0.91/0.89/0.95/0.98	0.21

Abbreviations: AIC = Akaike information criterion, BIC = Bayesian information criterion.

### 2.4 Associations Between LPA Classes and MASCS Measures and Interaction Analyses

The further analyses were performed using R, version 4.2.2. The libraries utilized were ggplot2 (Wickham 2016) and boot (Davison et al. 2003). *p*‐values (two‐tailed) less than 0.05 were considered to indicate statistical significance. The beta coefficients (*b*) and 95% confidence intervals (CI) were calculated. The distribution of the model's residuals was skewed, so bias‐corrected and accelerated (BCa) (Efron 1987) 95% bootstrap confidence intervals were also calculated (based on 5000 bootstrap samples) for the regression parameters.

First, the associations between LPA classes and MASCS subscales were examined using the following linear models:

(A) MASCS subscale~intercept + LPA class + PIQ + education, where the dependent variable is one of the MASCS subscales, LPA class is a binary variable, PIQ is a continuous variable, and mother's education is a categorical variable.

Second, the associations between age at which child entered ECEC and MASCS scores were examined using a linear model:

(B) MASCS subscale~intercept + age at which child entered ECEC + PIQ + education, where the age at which the child entered ECEC was treated as a continuous variable.

The interaction between age at which child entered ECEC and the LPA class was added to Model B to explore any moderation effects if the LPA class showed statistical significance in its association with MASCS in Model A.

Third, the associations between mothers' social networks and MASCS were examined using a linear model:

(C) MASCS subscale~intercept + social network subscale + PIQ + education, where the social networks are one of the five subscales (Table 2). Interactions between social network subscales and LPA class were added to Model C to examine the moderation effect if the LPA class showed statistical significance in its association with MASCS in Model A.

**TABLE 2:** Descriptive statistics of the participants and study variables.

Sample *N*	528
Child age (years), mean (SD)	5.02 (0.08)
Child sex (girls), *N* (%)	233 (44.1%)
Age at the time child entered ECEC, mean (SD)^a^	30 (16.1)
9–24 months, *N*	224
25 months or older, *N*	231
MASCS measures, mean (SD)	
Prosocial behavior	26.2 (3.1)
Cooperation skills	16.3 (2.2)
Empathy	9.9 (1.3)
Antisocial behavior	13.9 (3.1)
Impulsivity	6.7 (1.8)
Disruptiveness	7.2 (1.9)
Performance intelligence (PIQ), mean (SD), interquartile range^b^	102.35 (16.24) 91–112
Maternal characteristics when the child was 5 years old	
Age (years), mean (SD)^c^	36.6 (4.26)
Satisfaction to income (Scale 0–15)^c^	10.0 (3.24)
Employment status, *N* (%)^c^	
Working full‐time	293 (65.1)
Working part‐time	54 (12.0)
Unemployed	12 (2.7)
Stay‐at‐home parent	51 (11.3)
Studying	20 (4.4)
Other	20 (4.4)
Maternal education, *N* (%)^c^	
High school/vocational education	127 (24.8%)
Applied university	147 (28.7%)
University degree	239 (46.6%)
Mother's social networks, mean (SD)^d^	
Connections to friends and relatives	4.03 (0.9)
Support received from child's grandparents	3.76 (1.2)
Help received for childcare when needed	4.02 (1.1)
Cooperation with neighbors	2.86 (1.2)
Taking care of the child together with child's other parent	3.95 (1.1)

^a^ *n* = 455.

^b^ *n* = 526.

^c^ *n* = 449–513.

^d^ *n* = 512–513.

## 3 Results

Table 2 describes the characteristics of the participants. All the participants were Finnish and maternal origin and native language were primarily Finnish.

### 3.1 Latent Profiles of Maternal Symptoms and Child Socioemotional Outcomes (RQ1)

**FIGURE 2:**
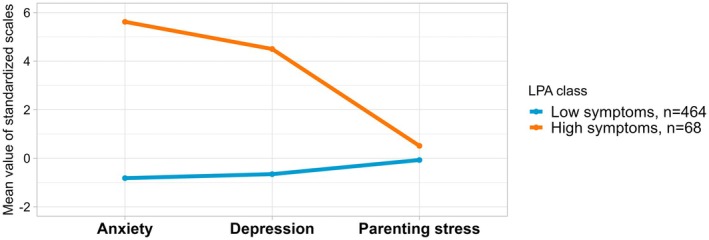
The latent profiles of cumulative maternal psychological distress from 3 months to 4 years postpartum were defined as symptoms of anxiety, depression, and parenting stress.

1. The LPA identified two latent profile groups related to maternal cumulative psychological distress. Most of the mothers (*n* = 464) belonged to the group with cumulatively low anxiety, low depression, and rather low parenting stress symptoms. A smaller number of mothers (*n* = 68) belonged to the group with high cumulative anxiety and high depression but rather low parenting stress symptoms (Figure 2).

**FIGURE 3:**
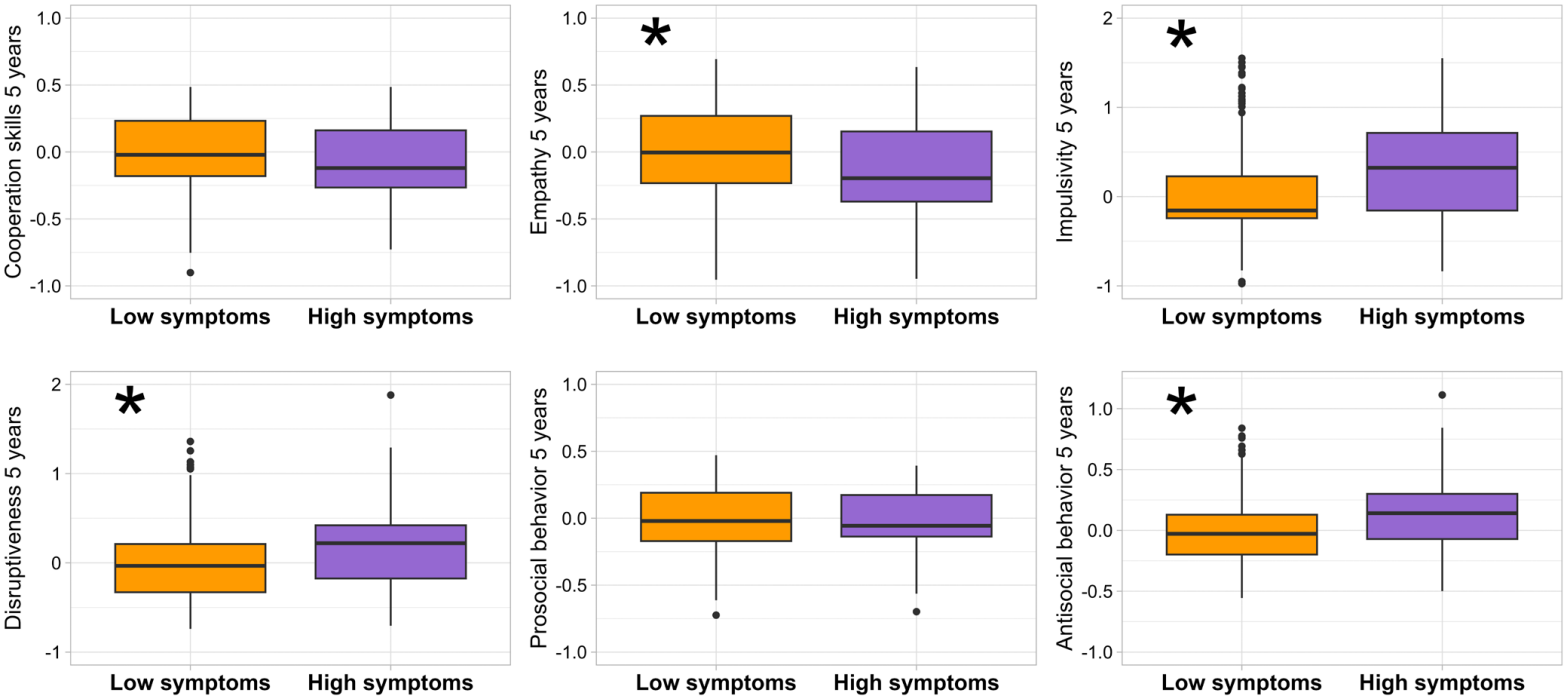
Associations between maternal psychological distress symptoms and children's cooperating skills and empathy (prosocial behavior) and between impulsivity and disruptiveness (antisocial behavior) (*statistically significant associations).

2 Higher maternal symptoms associated with child's higher impulsivity and higher disruptiveness, together forming an antisocial dimension of social competence. Higher maternal symptoms were also associated with lower empathy, which is part of the prosocial dimension of social competence (Figure 3; Table 3).

**TABLE 3:** Associations between LPA classes and MASCS scores.

Dependent variable		*b*	95% CI	*p*	95% BCa CI
Cooperation skills	Intercept	0.082	−0.073 to 0.237	0.301	−0.066 to 0.236
	LPA^a^	−0.072	−0.146 to 0.002	0.057	−0.154 to 0.007
	PIQ^c^	−0.001	−0.002 to 0.001	0.363	−0.002 to 0.001
	Education (mid)^b^	0.041	−0.025 to 0.107	0.222	−0.028 to 0.106
	Education (high)^b^	−0.007	−0.067 to 0.054	0.823	−0.071 to 0.054
Empathy	Intercept	0.084	−0.118 to 0.286	0.414	−0.114 to 0.286
	LPA^a^	−0.114	−0.211 to −0.017	0.021*	−0.215 to −0.014
	PIQ^c^	−0.001	−0.002 to 0.001	0.578	−0.003 to 0.001
	Education (mid)^b^	0.044	−0.042 to 0.130	0.311	−0.044 to 0.130
	Education (high)^b^	−0.025	−0.104 to 0.054	0.539	−0.110 to 0.055
Impulsivity	Intercept	−0.011	−0.297 to 0.275	0.940	−0.276 to 0.270
	LPA^a^	0.321	0.184 to 0.458	< 0.001*	0.147 to 0.486
	PIQ^c^	−0.0004	−0.003 to 0.002	0.785	−0.003 to 0.002
	Education (mid)^b^	−0.014	−0.136 to 0.108	0.821	−0.135 to 0.108
	Education (high)^b^	0.004	−0.108 to 0.115	0.949	−0.109 to 0.111
Disruptiveness	Intercept	−0.025	−0.255 to 0.206	0.834	−0.238 to 0.207
	LPA^a^	0.181	0.071 to 0.292	0.001*	0.044 to 0.325
	PIQ^c^	−0.0002	−0.002 to 0.002	0.844	−0.002 to 0.002
	Education (mid)^b^	−0.007	−0.105 to 0.091	0.890	−0.103 to 0.093
	Education (high)^b^	0.023	−0.067 to 0.112	0.622	−0.072 to 0.113
Prosocial behavior (consisted of cooperation and empathy)	Intercept	0.022	−0.103 to 0.147	0.730	−0.103 to 0.152
	LPA^a^	−0.013	−0.072 to 0.047	0.681	−0.072 to 0.044
	PIQ^c^	−0.0001	−0.001 to 0.001	0.872	−0.001 to 0.001
	Education (mid)^b^	0.016	−0.037 to 0.069	0.556	−0.036 to 0.069
	Education (high)^b^	−0.023	−0.072 to 0.025	0.345	−0.075 to 0.024
Antisocial behavior (consisted of impulsivity and disruptiveness)	Intercept	−0.017	−0.163 to 0.128	0.813	−0.155 to 0.121
	LPA^a^	0.127	0.058 to 0.197	< 0.001*	0.040 to 0.217
	PIQ^c^	−0.0001	−0.002 to 0.001	0.848	−0.001 to 0.001
	Education (mid)^b^	−0.008	−0.069 to 0.054	0.807	−0.067 to 0.054
	Education (high)^b^	0.016	−0.041 to 0.073	0.577	−0.040 to 0.072

*Note:* * as a mark for statistically significant values.

^a^ The reference level is low maternal symptoms.

^b^ The reference level is low.

^c^ Performance intelligence (PIQ).

### 3.2 Associations Between ECEC Participation, Mothers' Social Networks and Child Socioemotional Outcomes (RQ2)

**FIGURE 4:**
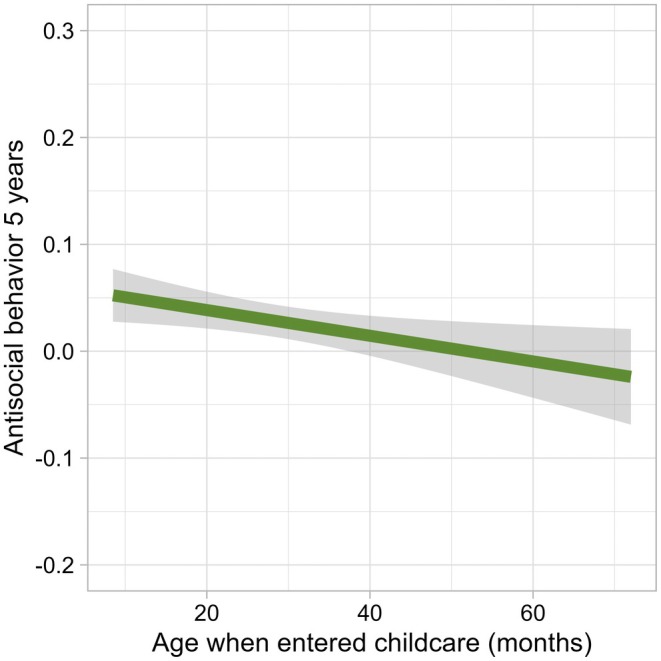
Association between ages at which child entered ECEC and antisocial behavior at the age of 5 years.

**FIGURE 5:**
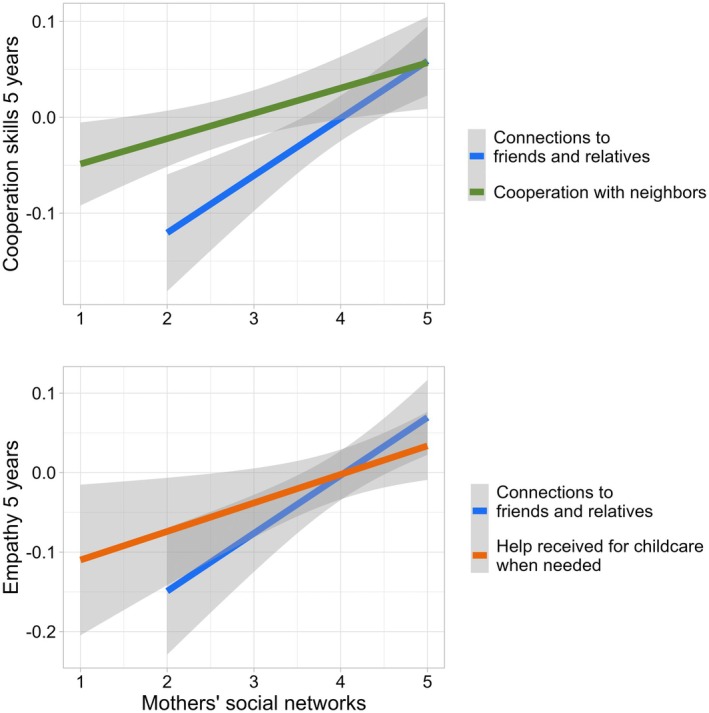
Associations between mother's social networks and child's cooperation skills and empathy at the age of 5 years.

3 The results also indicated that the younger the child entered ECEC, the more disruptive (*b* = −0.003, BCa CI [−0.005 to −0.0004], *p* = 0.024) and antisocial behavior (*b* = −0.002, BCa CI [−0.003 to −0.0001], *p* = 0.039) mothers reported at the child age of 5 years (Figure 4). The current results also showed that mothers' social networks were associated with children's higher empathy and cooperation skills. In particular, mothers' connection with friends and relatives (*b* = 0.07, BCa CI [0.03–0.11], *p* < 0.001) and help with childcare when needed (*b* = 0.04, BCa CI [0.01–0.07], *p* = 0.015) were associated with children's higher empathy. Moreover, mothers' connections with friends and relatives (*b* = 0.6, BCa CI [0.02–0.09], *p* < 0.001) and cooperation with neighbors (*b* = 0.02, BCa CI [0.01–0.04], *p* = 0.022) were associated with children's higher cooperation skills (Figure 5).

### 3.3 Moderating Role of ECEC Participation and Maternal Social Networks (RQ3)

4 We found no evidence for age at the time the child entered ECEC or the mother's social networks as moderators of the association between maternal symptoms and the child's prosocial or antisocial behavior (Table 4).

**TABLE 4:** Interactions between mothers' social networks, age at the time child entered ECEC, and LPA classes (the main effects are presented in Table 3).

Dependent variable	Interaction term	*b*	95% CI	*p*	95% BCa CI
Empathy	Soc 1^b^ × LPA^a^	0.028	−0.075 to 0.131	0.595	−0.112 to 0.072
	Soc 2^c^ × LPA^a^	0.005	−0.077 to 0.087	0.907	−0.077 to 0.062
	Soc 3^d^ × LPA^a^	−0.059	−0.146 to 0.028	0.185	−0.060 to 0.095
	Soc 4^e^ × LPA^a^	−0.017	−0.106 to 0.072	0.705	−0.036 to 0.081
	Soc 5^f^ × LPA^a^	0.046	−0.046 to 0.138	0.325	−0.072 to 0.074
	Age in ECEC^g^ × LPA^a^	−0.001	−0.009 to 0.007	0.780	−0.008 to 0.006
Impulsivity	Soc 1^b^ × LPA^a^	−0.098	−0.244 to 0.049	0.190	−0.038 to 0.191
	Soc 2^c^ × LPA^a^	0.013	−0.103 to 0.128	0.827	−0.079 to 0.110
	Soc 3^d^ × LPA^a^	0.027	−0.096 to 0.150	0.664	−0.112 to 0.087
	Soc 4^e^ × LPA^a^	−0.008	−0.133 to 0.117	0.900	−0.099 to 0.061
	Soc 5^f^ × LPA^a^	−0.001	−0.131 to 0.128	0.987	−0.071 to 0.122
	Age in ECEC^g^ × LPA^a^	−0.006	−0.017 to 0.006	0.314	−0.021 to 0.009
Disruptiveness	Soc 1^b^ × LPA^a^	−0.057	−0.175 to 0.062	0.348	−0.107 to 0.097
	Soc 2^c^ × LPA^a^	0.004	−0.090 to 0.098	0.934	−0.072 to 0.073
	Soc 3^d^ × LPA^a^	0.023	−0.076 to 0.123	0.644	−0.114 to 0.051
	Soc 4^e^ × LPA^a^	0.032	−0.070 to 0.134	0.540	−0.068 to 0.062
	Soc 5^f^ × LPA^a^	−0.008	−0.113 to 0.096	0.874	−0.022 to 0.136
	Age in ECEC^g^ × LPA^a^	−0.009	−0.019 to −0.0002	0.045	−0.019 to 0.001
Antisocial behavior	Soc 1^b^ × LPA^a^	−0.042	−0.117 to 0.032	0.267	−0.055 to 0.069
	Soc 2^c^ × LPA^a^	0.006	−0.053 to 0.065	0.839	−0.045 to 0.048
	Soc 3^d^ × LPA^a^	0.022	−0.040 to 0.085	0.486	−0.070 to 0.032
	Soc 4^e^ × LPA^a^	0.017	−0.047 to 0.082	0.594	−0.045 to 0.038
	Soc 5^f^ × LPA^a^	−0.006	−0.072 to 0.060	0.863	−0.018 to 0.080
	Age in ECEC^g^ × LPA^a^	−0.006	−0.011 to 0.0001	0.056	−0.012 to 0.001

^a^ The reference level is low maternal symptoms. All of the models included PIQ, mother's education, and the terms in the interactions (models b and c).

^b^ Connections to friends and relatives.

^c^ Support received from the child's grandparents.

^d^ Help received for childcare when needed.

^e^ Cooperation with neighbors.

^f^ Taking care of the child together with the child's other parent.

^g^ Age at the time the child entered ECEC.

## 4 Discussion

The aim of the present study was to investigate whether maternal cumulative psychological distress, including parenting stress, maternal anxiety, and depressive symptoms, from 3 months to 4 years postpartum was associated with a child's subsequent prosocial and antisocial behavior at the age of 4 years. In this study, we were able to investigate whether different maternal psychological distress symptoms co‐occur and accumulate during the postnatal period. Chronic and multiple types of maternal symptoms may have the most detrimental impact on child development. We further investigated whether a mother's social networks and the age at which a child entered ECEC were directly associated with the child's prosocial and antisocial behavior, and whether these factors moderated the associations between maternal psychological distress and children's behavioral outcomes. This study is important because it enhances our understanding of the risk and protective factors associated with a child's development. We focused not only on maternal well‐being but also on less frequently studied factors, such as maternal social networks and ECEC environments, and their potential protective role in children's socioemotional development.

The latent profile analysis identified two separable groups of maternal cumulative distress symptoms from 3 months to 4 years of age. Most of the mothers did not report any problems with their well‐being; hence, they belonged to the group with low anxiety and low depressive symptoms and rather low parenting stress. However, a smaller number of the mothers belonged to the group with chronically high anxiety and high depressive symptoms during the follow‐up period. Surprisingly, the level of parenting stress symptoms remained rather low in this group.

Maternal cumulative psychological distress was associated with higher impulsivity, higher disruptiveness, and lower empathy in children at the age of 5 years. This finding is in line with earlier research suggesting that maternal psychological distress is associated with adverse child outcomes (Behrendt et al. 2020; Goodman et al. 2011). A recent study further showed that maternal pre‐ and postnatal depressive and anxiety symptoms influence children's socioemotional competence and externalizing and internalizing problems from toddlerhood to preschool years indicating long‐term effects of maternal symptoms on child outcomes (Korja et al. 2024). Maternal depressive and anxiety symptoms have been shown to compromise the parent–child relationship and have adverse effects on parenthood (Liu et al. 2020). For instance, maternal depressive symptoms may limit maternal sensitivity to children, thus affecting the development of children's social skills (Feldman et al. 2009). Additionally, mothers with postnatal anxiety symptoms have reported lower parental self‐efficacy, less affectionate interaction, and less involvement with their child in everyday activities (Seymour et al. 2015). A systematic review by Jones et al. (2021) showed that mothers with a high level of anxiety more often showed overprotective parenting, which may limit a child's ability to cope with challenges. This finding is in line with the previous findings of Hakanen et al. (2019), suggesting that general anxiety symptoms are associated with higher maternal intrusiveness.

Nevertheless, it is important to note that parent–child interaction is bidirectional, and a child's characteristics also influence and shape that interplay (Sameroff 2009). Some previous studies have demonstrated bidirectional effects between maternal well‐being and child socioemotional development. For example, Lowthian et al. (2023) reported that children's emotional difficulties at age seven were predictive of subsequent increases in maternal mental health problems. Furthermore, within the same study, maternal distress symptoms were found to be associated with later elevations in children's emotional problems, indicating a bidirectional pattern of influence. Also, Verhagen et al. (2026) found bidirectional associations between maternal emotion regulation difficulties and child socioemotional problems during early childhood up to child age of 3 years. Additionally, child neuropsychiatric phenotypes, such as ADHD or autism spectrum symptoms, which are frequently intertwined with socioemotional difficulties, can increase parenting stress and further exacerbate parental psychological distress (Allmann et al. 2022; Roubinov et al. 2023).

In the present study, such bidirectional associations between maternal psychological distress and children's socioemotional problems could not be examined, as children's social and emotional outcomes were assessed using different measures across the follow‐up periods. Consequently, the analyses focused on social competence as measured by the MASCS at 5 years, and the longitudinal modeling of bidirectional effects using recommended methodological approaches (e.g., random‐intercept panel models, or cross‐lagged panel models) was not possible. Instead, the analyses addressed the one‐way effects of long‐term maternal symptoms and their associations with subsequent child developmental outcomes.

There are probably also other mechanisms that influence children's socioemotional development. Earlier research has shown that mothers who experience prenatal depression or anxiety are at increased risk of having children with behavioral difficulties. One possible mechanism involves the maternal hypothalamic–pituitary–adrenal (HPA) axis, which produces elevated levels of cortisol and may therefore affect children's brain development and regulatory capacity (Madigan et al. 2018).

These findings highlight the importance of detecting maternal depressive and anxiety symptoms as early as possible while offering appropriate support to ensure the optimal rearing environment for a child. Further research should also consider developmental trajectories and identify potential subgroups of mothers whose symptoms may remain stable or change over time as the child develops.

Earlier research has suggested that parents with depressive symptoms also experience more parenting stress (Lim and Shim 2021). However, in the present study, both groups identified in the LPA showed rather low parenting stress levels, regardless of the amount of depressive and anxiety symptoms. This discrepancy may be due to differing sources of depression and parenting stress. Parenting stress specifically arises from the demands of parenting and a lack of social support (Louie et al. 2017; Östberg et al. 1997). It is possible that the mothers in our sample had access to resources that helped them manage parenting stress, even if they experienced depressive or anxiety symptoms. The study population was highly educated, which may have contributed to their ability to seek and receive support and to maintain social networks that are important for well‐being and coping.

Additionally, parenting stress in our study was measured relatively early—at 3 and 12 months of age—whereas it may increase as children grow from infancy into toddlerhood and preschool age. Previous research suggests that parental satisfaction tends to be highest during pregnancy and the early years of the child's life, then decreasing over time (Pollmann‐Schult 2014). As children grow, parents may encounter greater challenges and pressures. Moreover, maternal depressive and anxiety symptoms may be more stable over time than parenting stress. In the present study, parenting stress was measured only during the first year of the child's life, whereas depressive and anxiety symptoms were assessed consecutively until the child reached age four.

We also examined the direct associations between mothers' social networks, the age at which the child entered ECEC, and the child's prosocial and antisocial behavior. The results indicated that mothers with richer social networks reported child's higher empathy and cooperation skills, which are subscales of prosocial behavior as measured by the MASCS. Specifically, mothers' connections with friends and relatives, help with childcare when needed, and cooperation with neighbors were positively associated with children's prosocial behavior. This finding aligns with previous studies suggesting that a family's social networks may form an important protective factor in a child's development (Hong and Liu 2021; Park and Lee 2022).

There are several mechanisms through which social networks may facilitate children's social development. Social networks and support may increase parental resources and lead to more positive parenting practices, which likely have a direct effect on a child's well‐being and favorable development (Liu et al. 2020). Moreover, family's contacts with other people shape children's social environments by increasing opportunities to learn cooperation and social skills. In this way, parental networks may also have an important direct effect on the number of children's friendships and the frequency and quality of peer relations (Neitola et al. 2023).

Despite the positive associations between mothers' social networks and children's prosocial behavior, we found no moderation effect of social networks on the association between maternal psychological distress and children's antisocial behavior. This was contrary to our hypothesis that social networks would protect children from the effects of maternal psychological distress and would be particularly beneficial for those children experiencing adversity in their family environment. Previous research suggests that social networks play an important role when there is an added burden or parenting stress in the family (Hong and Liu 2021; Park and Lee 2022). The lack of a moderation effect may be due to our inability to evaluate how supportive mothers perceived these social networks. It is also possible that we had insufficient statistical power in this study. The number of mothers with symptoms was relatively small, although it was consistent with previous findings indicating that the prevalence of postnatal depressive symptoms ranges from 13% to 19% (O'Hara and McCabe 2013) and anxiety from 13% to 40% (Field 2018).

Nevertheless, it is important to highlight that these exosystem‐level social networks indirectly influenced children's experiences and had positive effects on their prosocial development. Relatives, friends, and neighbors appear to be valuable resources for families with young children, and close cooperation with them contributes positively to children's socioemotional development (Nagy et al. 2022). Thus, based on our findings, it is essential to promote social networks, find ways to reduce loneliness among families with young children, and provide children with opportunities to build cooperation and prosocial skills.

Finally, in contrast to our hypothesis, we found no moderating effect of ECEC participation. An early start in ECEC did not appear to protect children from the negative effects of maternal long‐term psychological distress. This result may be caused from the methodological limitations in our study. We had information only on the age at which children entered ECEC, but not a comparison group of children who did not participate in ECEC at all. Including such a comparison group of children could have clarified whether ECEC participation would weaken the associations between maternal psychological distress symptoms and child socioemotional problems. Based on previous research, we expected that ECEC participation would be particularly beneficial for children whose mothers had psychological distress (Charrois et al. 2017).

Contrary to expectations, our results showed that the earlier a child started in ECEC, the more disruptive behavior was reported by mothers at age five. Previous research on this topic has been somewhat contradictory, with both positive and negative effects of ECEC observed (NICHD 2003; Rey‐Guerra et al. 2023). Some studies have demonstrated positive outcomes, such as improved learning abilities and social competence (Esping‐Andersen et al. 2012; Wahler et al. 2017), as well as a protective effect against maternal depressive symptoms (Charrois et al. 2017). ECEC has also been found to be particularly beneficial for disadvantaged children and children from lower socioeconomic backgrounds (Berry et al. 2016). However, research also reports higher levels of externalizing and internalizing problems in children who began ECEC at earlier ages compared to those with less ECEC experience (NICHD 2003). It is important to note that the effectiveness and potential of ECEC are highly influenced by the social policies of each country, which guide families' decision‐making (OECD 2024). Therefore, comparisons between different countries can be challenging.

Based on our results, it is difficult to conclude why mothers reported more behavioral problems at the age of 5 years in children who started ECEC at younger ages. The quality of ECEC in Finland has been assessed as very high by both parents and teachers (Heikka et al. 2021). Nevertheless, there may be some differences between individual ECEC units, and we were unable to collect quality measures from the childcare centers where the children were participating. It is possible that large groups and insufficient support from caregivers may contribute to disruptive behavior in children (Burchinal et al. 2010).

Additionally, children's disruptive behavior at home could be a normal reaction to long days in center‐based ECEC without their parents. An earlier study by Drugli et al. (2023) showed that parents described toddlers as tired and clingy after childcare days, particularly when starting ECEC for the first time. We did not have reports on how many children participated part‐time in ECEC. Part‐time care might be a better option for some of the youngest children. However, most children in Finland spend 7 to 9 h daily in ECEC because most parents work full‐time. Furthermore, we did not have teachers' reports on children's behavior in ECEC. We do not know whether these children exhibited disruptive behavior in ECEC as well or only at home. More research is warranted to determine how different types of childcare and their quality influence children's development and well‐being.

### 4.1 Limitations and Future Directions

The present study has numerous strengths, such as its large sample size and longitudinal study design with multiple measures of maternal depression, anxiety, and parenting stress symptoms. However, there are also limitations that should be taken into consideration. In this study, the maternal cumulative latent depressive, anxiety, and parenting stress symptoms described the mean values of the symptoms in the postnatal period until the child reached the age of 4 years. However, our analysis method did not take into account which age of the child the mother had highest or lowest symptoms. It is possible that there is a sensitive period in child development in which maternal symptoms have a more prominent effect on developmental outcomes (Bagner et al. 2010). Future studies should analyze maternal distress and child socioemotional outcomes both cross‐sectionally and longitudinally and identify subgroups of mothers whose symptoms may change across child development. Future research should also consider bidirectional pathways between maternal psychological distress symptoms and child development, as there is empirical evidence that child and parental characteristics interact and influence one another over time.

In the current study, we did not have a validated instrument to measure the quality of maternal social networks, although the questions were adapted based on previous research on social support (see e.g., Kalland et al. 2022). Upcoming research should consider broader measures of social networks that also account for mothers' own experiences of this support (Sourander et al. 2025). Future studies should also consider paternal social networks and their supportive effect for child development as well as the father's own well‐being and family dynamics.

We were not able to collect any quality measures from the ECEC centers. Future studies should consider quality factors in ECEC centers, particularly within groups of the youngest children who may need the most support for their emotion regulation. We did not have reports of child behavior from the childcare teachers and therefore were unable to know whether the child's behavioral problems appeared only at home or also in ECEC. Future studies should consider various environmental factors using different methods, such as observation and interviews, and combine them with information on children's behavior and family factors.

Finally, we only had maternal reports of children's social competence, behavioral problems, and mothers' own symptoms, which has certain limitations. Previous research has suggested that mothers with depressive symptoms tend to evaluate their children's behavior more negatively (Östberg and Hagekull 2013). It would have been important to obtain both parents' reports of their children's behavior. Additionally, fathers' or other spouses' reports of their own depressive and anxiety and parenting stress symptoms would have broadened our understanding of the family dynamics. Recent findings indicate that fathers play an important role in a child's socioemotional development, independent of maternal influences. Paternal distress symptoms may also have a distinct impact on both child development and family functioning (Zecchinato et al. 2025). Additionally, earlier studies have suggested that one parent's parenting stress may increase the other parent's stress (Kerstis et al. 2016). However, at the family level, the higher psychosocial well‐being of one parent seems to protect the child, even if the other parent is experiencing depressive symptoms (Panula et al. 2020). Further research should also consider broader environmental stressors, such as socioeconomic status, employment conditions and relationship satisfaction, which strongly influence parental well‐being and child development. Early detection of problems is crucial, as is providing the necessary support for families to ensure the optimal development of the child.

### 4.2 Conclusion

The present study suggests that maternal long‐term postnatal depressive and anxiety symptoms are associated with higher antisocial and lower prosocial behaviors in children at age five. Contrary to our expectations, an early start in ECEC or mothers' social networks did not have a protective effect against the influence of maternal symptoms on children's prosocial or antisocial behavior. However, mothers' supportive social networks, such as cooperation with friends, neighbors, and relatives, as well as help received for childcare, were beneficial for all children and associated with higher prosocial behaviors, including higher empathy and improved cooperation skills. Social networks are important resources for families with young children. They also provide increased social interaction opportunities for children whose parents' own resources are limited. Therefore, at a societal level, it is important to consider how to foster cooperative infrastructure, reduce loneliness, and enable the development of diverse social networks for families with young children.

## Author Contributions

All authors are responsible for the contents of the article and had authority over manuscript preparation. **Katja Tervahartiala:** conceptualization, writing – original draft, data analysis, project administration. **Eeva‐Leena Kataja:** conceptualization, data analysis, writing – review and editing, funding acquisition. **Laura Perasto:** data analysis, writing – review and editing. **Niina Junttila:** conceptualization, data analysis, writing – review and editing, funding acquisition. **Marjukka Pajulo:** writing – review and editing. **Hasse Karlsson:** project administration, funding acquisition, writing – review and editing. **Noona Kiuru:** conceptualization, writing – review and editing, funding acquisition. **Saara Nolvi:** conceptualization, writing – review and editing, funding acquisition. **Linnea Karlsson:** project administration, funding acquisition, writing – review and editing, conceptualization. **Riikka Korja:** project administration, funding acquisition, writing – review and editing, conceptualization.

## Funding

This research was funded by The Strategic Research Council (SRC) established within the Research Council of Finland (Nos. 372253, 372254, and 372256), The Research Council of Finland, Centre of Excellence in Learning Dynamics and Intervention Research (No. 346121), The Research Council of Finland (No. 346790) and the Signe and Ane Gyllenberg Foundation and the Jane and Aatos Erkko Foundation.

## Ethics Statement

The current study meets the ethical guidelines and was performed in accordance with the ethical standards of the 1964 Declaration of Helsinki and its later amendments. The Ethics Committee of the Hospital District of Southwest Finland has approved the research with protocol number ETMK: 137/1801/2013.

## Conflicts of Interest

The authors declare no conflicts of interest.

## Data Availability

The datasets to reproduce the analyses are not publicly available because of restrictions imposed by the Finnish law, and the study's ethical permissions do not allow sharing of the data used in this study. Requests to access the datasets should be directed to the Principal Investigator of this research project.

## References

[sjop70087-bib-0001] Äikäs, A. , H. Pesonen , N. Heiskanen , M. Syrjämäki , L. Aavikko , and E. Viljamaa . 2023. “Approaches to Collaboration and Support in Early Childhood Education and Care in Finland: Professionals' Narratives.” European Journal of Special Needs Education 38, no. 4: 528–542. 10.1080/08856257.2022.2127081.

[sjop70087-bib-0002] Allmann, A. E. S. , D. N. Klein , and D. C. Kopala‐Sibley . 2022. “Bidirectional and Transactional Relationships Between Parenting Styles and Child Symptoms of ADHD, ODD, Depression, and Anxiety Over 6 Years.” Development and Psychopathology 34, no. 4: 1400–1411. 10.1017/S0954579421000201. 34103100

[sjop70087-bib-0003] Bagner, D. M. , J. W. Pettit , P. M. Lewinsohn , and J. R. Seeley . 2010. “Effect of Maternal Depression on Child Behavior: A Sensitive Period?” Journal of the American Academy of Child & Adolescent Psychiatry 49, no. 7: 699–707. 10.1016/j.jaac.2010.03.012. 20610139 PMC2901251

[sjop70087-bib-0004] Behrendt, H. F. , M. Wade , L. Bayet , C. A. Nelson , and M. Bosquet Enlow . 2020. “Pathways to Social‐Emotional Functioning in the Preschool Period: The Role of Child Temperament and Maternal Anxiety in Boys and Girls.” Development and Psychopathology 32, no. 3: 961–974. 10.1017/S0954579419000853. 31345275 PMC6982599

[sjop70087-bib-0005] Berry, D. , C. Blair , M. Willoughby , P. Garrett‐Peters , L. Vernon‐Feagans , and W. R. Mills‐Koonce . 2016. “Household Chaos and Children's Cognitive and Socio‐Emotional Development in Early Childhood: Does Childcare Play a Buffering Role?” Early Childhood Research Quarterly 34: 115–127. 10.1016/j.ecresq.2015.09.003. 29720785 PMC5926246

[sjop70087-bib-0006] Bronfenbrenner, U. 1979. The Ecology of Human Development: Experiments by Nature and Design. Harvard University Press.

[sjop70087-bib-0007] Burchinal, M. , N. Vandergrift , R. Pianta , and A. Mashburn . 2010. “Threshold Analysis of Association Between Child Care Quality and Child Outcomes for Low‐Income Children in Pre‐Kindergarten Programs.” Early Childhood Research Quarterly 25, no. 2: 166–176. 10.1016/j.ecresq.2009.10.004.

[sjop70087-bib-0008] Charrois, J. , S. M. Côté , C. Japel , et al. 2017. “Child‐Care Quality Moderates the Association Between Maternal Depression and Children's Behavioural Outcome.” Journal of Child Psychology and Psychiatry 58, no. 11: 1210–1218. 10.1111/jcpp.12764. 28677114

[sjop70087-bib-0009] Cox, J. L. , J. M. Holden , and R. Sagovsky . 1987. “Detection of Postnatal Depression.” British Journal of Psychiatry 150, no. 6: 782. 10.1192/bjp.150.6.782. 3651732

[sjop70087-bib-0010] Davison, A. C. , D. V. Hinkley , and G. A. Young . 2003. “Recent Developments in Bootstrap Methodology.” Statistical Science 18, no. 2: 141–157.

[sjop70087-bib-0011] Derogatis, L. R. , R. S. Lipman , and L. Covi . 1973. “SCL‐90: An Outpatient Psychiatric Rating Scale—Preliminary Report.” Psychopharmacology Bulletin 9, no. 1: 13–28. 4682398

[sjop70087-bib-0012] Dong, S. , Q. Dong , and H. Chen . 2022. “Mothers' Parenting Stress, Depression, Marital Conflict, and Marital Satisfaction: The Moderating Effect of Fathers' Empathy Tendency.” Journal of Affective Disorders 299: 682–690. 10.1016/j.jad.2021.12.079. 34953927

[sjop70087-bib-0013] Drugli, M. B. , K. Nystad , S. Lydersen , and A. S. Brenne . 2023. “Do Toddlers' Levels of Cortisol and the Perceptions of Parents and Professional Caregivers Tell the Same Story About Transition From Home to Childcare? A Mixed Method Study.” Frontiers in Psychology 14: 1165788. 10.3389/fpsyg.2023.1165788. 37333593 PMC10272817

[sjop70087-bib-0014] Efron, B. 1987. “Better Bootstrap Confidence Intervals.” Journal of the American Statistical Association 82, no. 397: 8410. 10.1080/01621459.1987.10478410.

[sjop70087-bib-0015] Eisenberg, N. , and T. L. Spinrad . 2014. “Multidimensionality of Prosocial Behavior: Rethinking the Conceptualization and Development of Prosocial Behavior.” In Prosocial Development. A Multidimensional Approach, edited by L. M. Padilla‐Walker and G. Carlo , 17–39. Oxford University Press.

[sjop70087-bib-0016] Esping‐Andersen, G. , I. Garfinkel , W. J. Han , K. Magnuson , S. Wagner , and J. Waldfogel . 2012. “Child Care and School Performance in Denmark and the United States.” Children and Youth Services Review 34, no. 3: 576–589. 10.1016/j.childyouth.2011.10.010. 24163491 PMC3806146

[sjop70087-bib-0017] Falah‐Hassani, K. , R. Shiri , and C. L. Dennis . 2017. “The Prevalence of Antenatal and Postnatal Co‐Morbid Anxiety and Depression: A Meta‐Analysis.” Psychological Medicine 47, no. 12: 2041–2053. 10.1017/S0033291717000617. 28414017

[sjop70087-bib-0018] Feldman, R. , A. Granat , C. Pariente , H. Kanety , J. Kuint , and E. Gilboa‐Schechtman . 2009. “Maternal Depression and Anxiety Across the Postpartum Year and Infant Social Engagement, Fear Regulation, and Stress Reactivity.” Journal of the American Academy of Child and Adolescent Psychiatry 48, no. 9: 919–927. 10.1097/CHI.0b013e3181b21651. 19625979

[sjop70087-bib-0019] Field, T. 2018. “Postnatal Anxiety Prevalence, Predictors and Effects on Development: A Narrative Review.” Infant Behavior and Development 51: 24–32. 10.1016/j.infbeh.2018.02.005. 29544195

[sjop70087-bib-0020] Geiser, C. 2013. Methodology in the Social Sciences. Data Analysis With Mplus. Guilford Press.

[sjop70087-bib-0021] Glasheen, C. , G. A. Richardson , and A. Fabio . 2010. “A Systematic Review of the Effects of Postnatal Maternal Anxiety on Children.” Archives of Women's Mental Health 13, no. 1: 61–74. 10.1007/s00737-009-0109-y. PMC3100191 19789953

[sjop70087-bib-0022] Goodman, S. H. , M. H. Rouse , A. M. Connell , M. R. Broth , C. M. Hall , and D. Heyward . 2011. “Maternal Depression and Child Psychopathology: A Meta‐Analytic Review.” Clinical Child and Family Psychology Review 14, no. 1: 1–27. 10.1007/s10567-010-0080-1. 21052833

[sjop70087-bib-0023] Hakanen, H. , M. Flykt , E. Sinervä , et al. 2019. “How Maternal Pre‐ and Postnatal Symptoms of Depression and Anxiety Affect Early Mother‐Infant Interaction?” Journal of Affective Disorders 257: 83–90. 10.1016/j.jad.2019.06.048. 31299408

[sjop70087-bib-0024] Heikka, J. , E. Fonsén , M. Mäntyjärvi , L. Kiuru , K. Suhonen , and L. Heikonen . 2021. “Evaluating Quality in Finnish Early Childhood Education.” In Quality Improvement in Early Childhood Education: International Perspectives on Enhancing Learning Outcomes, edited by S. Garvis and H. L. Taguchi , 21–44. Springer International Publishing. 10.1007/978-3-030-73182-3_2.

[sjop70087-bib-0025] Hohls, J. K. , H.‐H. König , E. Quirke , and A. Hajek . 2021. “Anxiety, Depression and Quality of Life—A Systematic Review of Evidence From Longitudinal Observational Studies.” International Journal of Environmental Research and Public Health 18, no. 22: 12022. 10.3390/ijerph182212022. 34831779 PMC8621394

[sjop70087-bib-0026] Hong, X. , and Q. Liu . 2021. “Parenting Stress, Social Support and Parenting Self‐Efficacy in Chinese Families: Does the Number of Children Matter?” Early Child Development and Care 191, no. 14: 2269–2280. 10.1080/03004430.2019.1702036.

[sjop70087-bib-0027] Huang, W. , S. Weinert , J. Von Maurice , and M. Attig . 2022. “Specific Parenting Behaviors Link Maternal Education to Toddlers' Language and Social Competence.” Journal of Family Psychology 36, no. 6: 998–1009. 10.1037/fam0000950. 35025535

[sjop70087-bib-0028] Jones, L. B. , B. A. Hall , and E. J. Kiel . 2021. “Systematic Review of the Link Between Maternal Anxiety and Overprotection.” Journal of Affective Disorders 295: 541–551. 10.1016/j.jad.2021.08.065. 34509069 PMC8551038

[sjop70087-bib-0029] Junttila, N. , M. Voeten , A. Kaukiainen , and M. Vauras . 2006. “Multisource Assessment of Children's Social Competence.” Educational and Psychological Measurement 66, no. 5: 874–895. 10.1177/0013164405285546.

[sjop70087-bib-0030] Kalland, M. , S. Salo , L. Vincze , et al. 2022. “Married and Cohabiting Finnish First‐Time Parents: Differences in Wellbeing, Social Support and Infant Health.” Social Sciences 11, no. 4: 181. 10.3390/socsci11040181.

[sjop70087-bib-0031] Karlsson, L. , M. Tolvanen , N. M. Scheinin , et al. 2018. “Cohort Profile: The FinnBrain Birth Cohort Study (FinnBrain).” International Journal of Epidemiology 47: 15–16. 10.1093/ije/dyx173. 29025073

[sjop70087-bib-0032] Kerstis, B. , E. Nohlert , J. Öhrvik , and M. Widarsson . 2016. “Association Between Depressive Symptoms and Parental Stress Among Mothers and Fathers in Early Parenthood: A Swedish Cohort Study.” Upsala Journal of Medical Sciences 121, no. 1: 60–64. 10.3109/03009734.2016.1143540. 26947219 PMC4812059

[sjop70087-bib-0033] Kochenderfer‐Ladd, B. , and G. W. Ladd . 2019. “Peer Relationships and Social Competence in Early Childhood.” In Handbook of Research on the Education of Young Children, edited by O. N. Saracho , 4th ed., 32–42. Routledge. 10.4324/9780429442827.

[sjop70087-bib-0034] Korja, R. , S. Nolvi , N. M. Scheinin , et al. 2024. “Trajectories of Maternal Depressive and Anxiety Symptoms and Child's Socio‐Emotional Outcome During Early Childhood.” Journal of Affective Disorders 349: 625–634. 10.1016/j.jad.2023.12.076. 38184113

[sjop70087-bib-0035] Kulic, N. , J. Skopek , M. Triventi , and H. Blossfeld . 2017. “Childcare, Early Education, and Social Inequality: Perspectives for a Cross‐National and Multidisciplinary Study.” In Childcare, Early Education and Social Inequality: An International Perspective, edited by H.‐P. Blossfeld , 3–28. Edward Elgar Publishing Limited. 10.4337/9781786432094.00008.

[sjop70087-bib-0036] Lim, S. A. , and S. Y. Shim . 2021. “Effects of Parenting Stress and Depressive Symptoms on Children's Internalizing and Externalizing Problems.” Journal of Child and Family Studies 30, no. 4: 989–1001. 10.1007/s10826-021-01929-z.

[sjop70087-bib-0037] Liu, S. , F. Zhai , and Q. Gao . 2020. “Parental Stress and Parenting in Chinese Immigrant Families: The Mediating Role of Social Support.” Child & Family Social Work 25, no. S1: 135–148. 10.1111/cfs.12734.

[sjop70087-bib-0038] Louie, A. D. , L. D. Cromer , and J. O. Berry . 2017. “Assessing Parenting Stress: Review of the Use and Interpretation of the Parental Stress Scale.” Family Journal 25, no. 4: 359–367. 10.1177/1066480717731347.

[sjop70087-bib-0039] Lovejoy, M. C. , P. A. Graczyk , E. O'Hare , and G. Neuman . 2000. “Maternal Depression and Parenting Behavior: A Meta‐Analytic Review.” Clinical Psychology Review 20, no. 5: 561–592. 10.1016/s0272-7358(98)00100-7. 10860167

[sjop70087-bib-0040] Lowthian, E. , S. Bedston , S. M. Kristensen , et al. 2023. “Maternal Mental Health and Children's Problem Behaviours: A Bi‐Directional Relationship?” Research on Child and Adolescent Psychopathology 51: 1611–1626. 10.1007/s10802-023-01086-5. 37400731 PMC10628040

[sjop70087-bib-0041] Madigan, S. , H. Oatley , N. Racine , et al. 2018. “A Meta‐Analysis of Maternal Prenatal Depression and Anxiety on Child Socioemotional Development.” Journal of the American Academy of Child and Adolescent Psychiatry 57, no. 9: 645–657.e8. 10.1016/j.jaac.2018.06.012. 30196868

[sjop70087-bib-0042] Minedu . 2025. “Early Childhood Education and Care—OKM—Ministry of Education and Culture, Finland.” https://okm.fi/en/early‐childhood‐education‐and‐care.

[sjop70087-bib-0043] Morales, M. F. , L.‐C. Girard , A. Raouna , and A. MacBeth . 2023. “The Association of Different Presentations of Maternal Depression With Children's Socio‐Emotional Development: A Systematic Review.” PLOS Global Public Health 3, no. 2: e0001649. 10.1371/journal.pgph.0001649. 36963088 PMC10021281

[sjop70087-bib-0044] Muthén, B. 2003. “Statistical and Substantive Checking in Growth Mixture Modeling: Comment on Bauer and Curran (2003).” Psychological Methods 8: 369–377. 14596497 10.1037/1082-989X.8.3.369

[sjop70087-bib-0045] Muthén, L. K. , and B. O. Muthén . 2010. Mplus User's Guide: Statistical Analysis With Latent Variables: User's Guide. Muthén & Muthén.

[sjop70087-bib-0046] Nagy, E. , S. Moore , P. P. Silveira , M. J. Meaney , R. D. Levitan , and L. Dubé . 2022. “Low Socioeconomic Status, Parental Stress, Depression, and the Buffering Role of Network Social Capital in Mothers.” Journal of Mental Health 31, no. 3: 340–347. 10.1080/09638237.2020.1793118. 32691647

[sjop70087-bib-0047] Neitola, M. , P. Af Ursin , and P. Pihlaja . 2023. “Explaining Children's Social Relationships in Early Childhood: The Role of Parental Social Networks.” European Early Childhood Education Research Journal 32: 1–16. 10.1080/1350293X.2023.2257912.

[sjop70087-bib-0048] NICHD . 2003. “Does Amount of Time Spent in Child Care Predict Socioemotional Adjustment During the Transition to Kindergarten?” Child Development 74, no. 4: 976–1005. 10.1111/1467-8624.00582. 12938694

[sjop70087-bib-0049] Nylund, K. L. , T. Asparouhov , and B. Muthen . 2007. “Deciding on the Number of Classes in Latent Class Analysis and Growth Mixture Modeling: A Monte Carlo Simulation Study.” Structural Equation Modeling: A Multidisciplinary Journal 14, no. 4: 535–569.

[sjop70087-bib-0050] OECD . 2024. “OECD Family Database—OECD.” http://www.oecd.org/els/family/database.htm.

[sjop70087-bib-0051] O'Hara, M. W. , and J. E. McCabe . 2013. “Postpartum Depression: Current Status and Future Directions.” Annual Review of Clinical Psychology 9: 379–407. 10.1146/annurev-clinpsy-050212-185612. 23394227

[sjop70087-bib-0052] Östberg, M. , and B. Hagekull . 2013. “Parenting Stress and External Stressors as Predictors of Maternal Ratings of Child Adjustment.” Scandinavian Journal of Psychology 54, no. 3: 213–221. 10.1111/sjop.12045. 23480459

[sjop70087-bib-0053] Östberg, M. , B. Hagekull , and S. Wettergren . 1997. “A Measure of Parental Stress in Mothers With Small Children: Dimensionality, Stability and Validity.” Scandinavian Journal of Psychology 38, no. 3: 199–208. 10.1111/1467-9450.00028. 9309950

[sjop70087-bib-0054] Panula, V. , N. Junttila , M. Aromaa , P. Rautava , and H. Räihä . 2020. “Parental Psychosocial Well‐Being as a Predictor of the Social Competence of a Child.” Journal of Child and Family Studies 29, no. 11: 3004–3019. 10.1007/s10826-020-01790-6.

[sjop70087-bib-0055] Park, G.‐A. , and O. N. Lee . 2022. “The Moderating Effect of Social Support on Parental Stress and Depression in Mothers of Children With Disabilities.” Occupational Therapy International 2022: 1–8. 10.1155/2022/5162954. PMC8938151 35359427

[sjop70087-bib-0056] Parkes, A. , M. Green , and A. Pearce . 2021. “Can Centre‐Based Childcare Buffer Against the Negative Effects of Family Adversity on Child Socio‐Emotional Wellbeing?” European Journal of Public Health 31, no. 3: 474–481. 10.1093/eurpub/ckab006. 33550396 PMC7611253

[sjop70087-bib-0057] Pollmann‐Schult, M. 2014. “Parenthood and Life Satisfaction: Why Don't Children Make People Happy?” Journal of Marriage and Family 76, no. 2: 319–336. 10.1111/jomf.12095.

[sjop70087-bib-0058] Rey‐Guerra, C. , H. D. Zachrisson , E. Dearing , et al. 2023. “Do More Hours in Center‐Based Care Cause More Externalizing Problems? A Cross‐National Replication Study.” Child Development 94, no. 2: 458–477. 10.1111/cdev.13871. 36385691

[sjop70087-bib-0059] Rost, J. 2006. “Latent Class Analysis.” In Handbook of Psychological Assessment, edited by F. Petermann and M. Eid , 275–287. Hogrefe.

[sjop70087-bib-0060] Roubinov, D. , B. Don , R. Blades , and E. Epel . 2023. “Is It Me or My Child? The Association Between Maternal Depression and Children's Behavior Problems in Mothers and Their Children With or Without Autism.” Family Process 62: 737–753. 10.1111/famp.12810. 36017571

[sjop70087-bib-0061] Sameroff, A. , ed. 2009. The Transactional Model of Development: How Children and Contexts Shape Each Other. American Psychological Association. 10.1037/11877-000.

[sjop70087-bib-0062] Santos, A. J. , B. E. Vaughn , I. Peceguina , J. R. Daniel , and N. Shin . 2014. “Growth of Social Competence During the Preschool Years: A 3‐Year Longitudinal Study.” Child Development 85, no. 5: 2062–2073. 10.1111/cdev.12246. 24749549

[sjop70087-bib-0063] Seymour, M. , R. Giallo , A. Cooklin , and M. Dunning . 2015. “Maternal Anxiety, Risk Factors and Parenting in the First Post‐Natal Year.” Child: Care, Health and Development 41, no. 2: 314–323. 10.1111/cch.12178. 25074519

[sjop70087-bib-0064] Sourander, J. , S. Salo , M. L. Laakso , and M. Kalland . 2025. “Toddlers' Social‐Emotional Competence and Problem Behaviours: Associations With Parenting Stress and Social Support Among Finnish First‐Time Parents.” Early Child Development and Care 195, no. 12: 1316–1336. 10.1080/03004430.2025.2549108.

[sjop70087-bib-0065] Tong, L. , R. Shinohara , Y. Sugisawa , et al. 2010. “Relationship Between Children's Intelligence and Their Emotional/Behavioral Problems and Social Competence: Gender Differences in First Graders.” Journal of Epidemiology 20: S466–S471. 10.2188/jea.JE20090146. 20179377 PMC3920395

[sjop70087-bib-0066] Vanin, J. R. 2008. “Overview of Anxiety and the Anxiety Disorders.” In Anxiety Disorders. Current Clinical Practice. Humana Press. 10.1007/978-1-59745-263-2_1.

[sjop70087-bib-0067] Verhagen, C. , S. Duijndam , N. Kupper , P. Lodder , and M. Boekhorst . 2026. “A Cross‐Lagged Panel Model Examining the Longitudinal Associations Between Maternal Emotion Regulation Difficulties, Parenting Stress, and Child Socio‐Emotional Problems in Toddlerhood.” Infant Mental Health Journal: Infancy and Early Childhood 47: e70051. 10.1002/imhj.70051. PMC12719906 41053968

[sjop70087-bib-0068] Wahler, S. , S. Buchholz , and A. Breinholt . 2017. “Childcare Arrangements at Preschool Age and Later Child Outcomes in Denmark: The Role of Maternal Education and Type of Care.” In Childcare, Early Education and Social Inequality. An International Perspective, edited by H.‐P. Blossfeld , N. Kulic , J. Skopek , and M. Triventi , 249–267. Elgar Publishing. 10.4337/9781786432094.

[sjop70087-bib-0069] Wechsler, D. 1991. “Wechsler Intelligence Scale for Children—Revised.”

[sjop70087-bib-0070] Wickham, H. 2016. ggplot2: Elegant Graphics for Data Analysis. Springer. 10.1007/978-0-387-98141-3.

[sjop70087-bib-0071] Zecchinato, F. , Y. I. Ahmadzadeh , J. M. Kreppner , and P. J. Lawrence . 2025. “A Systematic Review and Meta‐Analysis: Paternal Anxiety and the Emotional and Behavioral Outcomes in Their Offspring.” Journal of the American Academy of Child & Adolescent Psychiatry 64, no. 2: 172–197. 10.1016/j.jaac.2024.04.005. 38697345

[sjop70087-bib-0072] Zimmerman, M. , C. Balling , I. Chelminski , and K. Dalrymple . 2018. “Understanding the Severity of Depression: Which Symptoms of Depression Are the Best Indicators of Depression Severity?” Comprehensive Psychiatry 87: 84–88. 10.1016/j.comppsych.2018.09.006. 30282058

